# Factors associated with clinical severity in scorpion envenomation: a retrospective cross-sectional study in western Mexico between 2021 and 2025

**DOI:** 10.3389/ftox.2026.1880720

**Published:** 2026-07-24

**Authors:** Héctor Andrés González-Ruiz, Selene Guadalupe Huerta-Olvera, José Alberto Plascencia-Jiménez, Erick Yoav Martínez-Gallegos, Jonathan Moisés Guzmán-Villalpando, Fanny Iraís Aviña-Galindo, Carla Arleth Ojeda-Vázquez, Eduardo Alfonso Hernández-Muñoz, Eugenio Vladimir Zavala-Sánchez, Hilda Vázquez-López, Alejandro Alagón, Christian Johana Baños-Hernández

**Affiliations:** 1 Doctoral Program in Pharmacology, Health Sciences University Center, University of Guadalajara, Guadalajara, Jalisco, Mexico; 2 Department of Medical and Life Sciences, Ciénega University Center, University of Guadalajara, Ocotlán, Jalisco, Mexico; 3 Department of Population Health, New York University School of Medicine, New York, NY, United States; 4 Tlajomulco University Center, University of Guadalajara, Tlajomulco de Zúñiga, Jalisco, Mexico; 5 Family Medicine Unit No. 181, Mexican Social Security Institute, Ixtlahuacán de los Membrillos, Jalisco, Mexico; 6 Epidemiology Department, General Zone Hospital No. 89, Instituto Mexicano del Seguro Social, Guadalajara, Jalisco, Mexico; 7 Department of Molecular Medicine and Bioprocesses, Institute of Biotechnology, National Autonomous University of Mexico, Cuernavaca, Morelos, Mexico; 8 Biomedical Sciences Research Institute, Health Sciences University Center, University of Guadalajara, Guadalajara, Jalisco, Mexico

**Keywords:** antivenom, centruroides, clinical severity, Mexico, scorpion envenomation, scorpion sting, toxicology, traditional remedies

## Abstract

**Introduction:**

Scorpion envenomation is a major public health concern in Mexico. Although Q9 most of the approximately 300,000 annual cases are resolved without complications, a subset present with moderate or severe manifestations, and around 35 deaths are reported each year. Identifying factors associated with greater clinical severity may improve clinical assessment and management. Our objective was to evaluate factors associated with clinical severity among scorpion envenomated patients in western Mexico.

**Methods:**

We conducted a retrospective crosssectional study of patients with scorpion envenomation treated at an emergency department in Mexico between 2021 and 2025. Multivariable regression models were used to examine the association of demographic characteristics, time to medical care, and prehospital practices with clinical severity.

**Results:**

Of 3,145 patients, 32% were mild, 60% moderate, and 8.2% severe. Delayed access to medical care was associated with higher odds of moderate or severe envenomation whereas older age and higher body weight were protective. Overall use of traditional remedies, mainly milk and garlic, was not associated with severity. However, oral garlic use was associated with increased odds of moderate or severe envenomation and more frequent upper pharyngeal symptoms.

**Discussion:**

These findings suggest that delays in care-seeking are consistently associated with more severe clinical presentation, whereas age-related factors may confer relative protection. The observed association with garlic use, likely reflects differences in care-seeking behavior or symptom perception rather than a direct biological effect. Interventions should prioritize reducing delays to medical evaluation and addressing prehospital practices linked to delayed presentation.

## 1 Introduction

Scorpions are found in most parts of the world; however, only certain countries have species that are considered medically important (Dunlop and Selden, 2013). Most medically significant scorpions are in the *Buthidae* family and are distributed in five of the six World Health Organization Regions: *Androctonus* and *Leiurus* (African, Eastern Mediterranean and European), *Buthus* (African, and Eastern Mediterranean), *Hottentotta* (South-East Asian), *Mesobuthus* and *Nebo* (Eastern Mediterranean), *Centruroides* and *Tytius* (The Americas). Tityus species are common in South America and the Caribbean, while *Centruroides* is found in Mexico, Central America, and the southwestern U.S. (Vasconez-Gonzalez et al., 2025; Hernández Muñoz et al., 2025a).

Scorpion envenomation remains a significant public health problem in Mexico, accounting for a substantial proportion of emergency department visits in endemic regions (Chippaux et al., 2020). Approximately 300,000 people seek medical attention each year for scorpion sting envenomation, and an estimated 35 to 50 fatalities occur annually (Sistema Nacional de Vigilancia Epidemiológica, 2024; Direccion General de Epidemiología, 2024; Sistema Nacional de Vigilancia Epidemiológica, 2025). Around 20 species of the genus *Centruroides* are considered medically important in the region, including *Centruroides limpidus, C. baergi, C. balsasensis, C. bonito, C. chamela, C. elegans, C. hirsutipalpus, C. infamatus, C. mascota, C. meisei, C. noxius, C. ornatus, C. sculpturatus, C. suffusus, C. tecomanus, and C. villegasi.* The venoms of these species are predominantly neurotoxic, causing a characteristic clinical syndrome driven by voltage-gated sodium channel modulation and autonomic dysfunction, typically manifested by neuromuscular hyperexcitability and parasympathetic features (Valdez-Velazquez et al., 2025; Godoy et al., 2021). This pattern differs from that observed in other medically important genera such as *Tityus, Androctonus,* and *Hottentotta*, where differences in toxin composition are more frequently associated with cardiotoxic effects and predominant sympathetic manifestations, resulting in distinct clinical patterns and severity profiles (Hernández-Muñoz and Zavala-Sánchez, 2024; Abroug et al., 2020; LoVecchio, 2026; Isbister and Bawaskar, 2014).

Some studies have shown that scorpion sting incidence exhibits marked seasonal variation and is strongly associated with climatic conditions, particularly ambient and minimum temperatures. In endemic regions of western Mexico, increases in temperature have been associated with higher sting incidence, suggesting that climatic factors play an important role in the epidemiology of scorpionism (Chowell et al., 2005). More recently, analyses conducted in Jalisco, identified a positive association between temperature and scorpion sting incidence, with an estimated increase of 12.7 reported cases per 1 °C rise in temperature (R^2^ = 0.48), highlighting the relevance of environmental factors in local transmission dynamics (Aviña-Galindo et al., 2025).

Despite meeting all World Health Organization criteria for recognition as a neglected tropical disease (e.g., high morbidity with severe health consequences, disproportionate impact on impoverished populations, complex epidemiological patterns, and significant barriers to public health control), scorpion envenomation remains unrecognized as such (Hernández Muñoz et al., 2025a). Clinical research on scorpion envenomation remains limited, and available evidence is highly region-specific, reflecting differences in scorpion genera, species, and health system contexts. Taken together, these factors highlight persistent gaps in the epidemiological and clinical understanding of scorpion envenomation globally.

The clinical course of scorpion envenomation is shaped by a combination of factors, including patient characteristics, scorpion species, underlying health conditions, and the timeliness of medical intervention (Klotz et al., 2021; Klotz et al., 2023; Chowell et al., 2006). According to Mexican clinical guidelines, envenomation severity is classified into three levels: mild, moderate, and severe (CENETEC. Guía de Práctica Clínica, 2015). Clinical severity reflects an increasing number and complexity of signs and symptoms, encompassing both their type and extent of systemic involvement.

Scorpion antivenom is the treatment of choice for scorpion envenomation (Abroug et al., 2020). Although different dosing regimens have been proposed, the administration of one to six vials of antivenom is generally accepted for the management of envenomed patients (CENETEC. Guía de Práctica Clínica, 2015; Food and drug administration, 2011; Silanes, 2023). Despite the widespread availability of effective antivenom therapy, moderate and severe envenomation continues to pose important clinical and health system challenges. Delays in access to antivenom, shortages of trained healthcare personnel, and limited resources in both urban and rural settings contribute to adverse outcomes in patients with severe envenomation (Chippaux et al., 2020).

Previous studies have suggested that both pediatric and older patients may be at increased risk of worse clinical outcomes following scorpion envenomation (Isbister and Bawaskar, 2014; Chowell et al., 2006). Although the importance of early antivenom administration has been consistently emphasized, considerably less attention has been paid to behavioral and prehospital factors that may indirectly influence clinical severity (Direccion General de Epidemiología, 2024; Klotz et al., 2021; Klotz et al., 2023).

In many endemic settings, patients or caregivers engage in prehospital practices, such as the use of traditional remedies, before seeking medical care (Lagunas-Flores and Lagunas-Jaimes, 2009). These behaviors are context-dependent and culturally embedded, reflecting not only local health beliefs and intergenerational knowledge, but also differences in healthcare accessibility and availability across communities. Their use may therefore represent adaptive responses to social, geographic, and healthcare-system constraints rather than solely treatment preferences, and their relationship with clinical severity remains incompletely understood. Rather than acting as direct determinants of toxicity, such practices may interact with established risk factors by altering symptom perception and delaying access to medical evaluation (Hernández-Muñoz et al., 2025b).

The research question addressed in this study was: which demographic, clinical, and prehospital factors are associated with increased clinical severity in scorpion envenomation in a large retrospective sample from western Mexico? We hypothesized that established predictors such as younger age, lower body weight, and longer time to medical care would be associated with greater clinical severity. Additionally, we hypothesized that prehospital practices, particularly the use of traditional remedies, may influence clinical severity, potentially through delayed access to medical care.

## 2 Methods

In this retrospective cross-sectional study, routinely collected clinical data from a referral emergency department of a Public Hospital in San Pedro Tlaquepaque, a municipality within the Guadalajara Metropolitan Area, Jalisco, Mexico, were used, covering the period from January 2021 to December 2025.

### 2.1 Study setting

The study was conducted in the Guadalajara Metropolitan Area, located in the state of Jalisco in Western Mexico. The Guadalajara Metropolitan Area comprises several municipalities surrounding Guadalajara, the capital city of Jalisco, and is one of the largest urban regions in Mexico (Instituto de Planeación y Gestión del Desarrollo, 2026). The region is characterized by warm and semi-warm subhumid climates with summer rainfall and includes both highly urbanized and peri-urban areas (Instituto Nacional de Estadística y Geografía, 2023). Jalisco is among the Mexican states with the highest burden of scorpion envenomation and is home to medically important species of the genus *Centruroides*, including *C. limpidus* and *C. tecomanus* (González-Santillán and Possani, 2018). The study hospital serves as a referral center for patients from the metropolitan area and surrounding regions.

### 2.2 Participants

All patients with confirmed scorpion envenomation, based on the current operational definitions described in the Manual of Standard Procedures for Epidemiological Surveillance of Scorpion Sting Envenomation following the coding of the ICD-11 (Code: PA78&XM9DM8), who presented during the study period were eligible for inclusion (Direccion General de Epidemiología, 2024; World Health Organization G, 2022). Case identification was based on the standardized national surveillance system. Patients with missing data for the primary outcome were included in descriptive analyses but excluded from multivariable regression models.

### 2.3 Data sources and measurement

Data were retrieved from physical medical records through manual review. A standardized data collection framework based on the national epidemiological reporting form “Estudio epidemiológico de caso de intoxicación por alacrán” (code SIPE 0601005-C1, Secretaría de Salud) was used to ensure consistency. Demographic, clinical, and prehospital variables were extracted from records completed at the time of patient care.

### 2.4 Variables and outcomes

The primary outcome was clinical severity, categorized as mild, moderate, or severe according to national guidelines (CENETEC. Guía de Práctica Clínica, 2015). Severity classification was assigned based on signs and symptoms documented at admission. To ensure consistency, the severity classification automatically recorded in the database was cross-checked against the clinical information documented in the medical record.

Severity was analysed using two complementary approaches: a binary classification, in which mild cases were defined by the presence of local manifestations only and moderate or severe cases by the presence of systemic signs and symptoms; and an ordinal classification preserving the three severity levels (mild, moderate, severe). Ordinal models were used as sensitivity analyses. Candidate variables included age, sex (reported by physician), body weight, time from sting to medical care, and prehospital use of traditional remedies. Time to medical care was calculated from recorded time of sting to time of hospital admission. Traditional remedies were evaluated both as individual and combined exposure for the most reported practices. Additionally, clinical signs and symptoms were analysed as individual manifestations and stratified according to severity and age to explore patterns of clinical presentation.

### 2.5 Statistical analysis

Descriptive statistics are reported as medians with interquartile ranges for continuous variables and as frequencies with percentages for categorical variables. Bivariate logistic regression models were used to estimate crude associations between selected variables and clinical severity.

Multivariable logistic regression models were fitted to examine the association between selected covariates and the odds of moderate or severe envenomation. Clinically relevant variables were included as covariates in the models. Continuous variables were analysed on their respective original scales.

Complete-case analysis was used for primary models. As a sensitivity analysis, multiple imputation was performed, yielding similar results. Additional sensitivity analyses included: substitution of age with body weight in multivariable models and ordinal logistic regression to evaluate clinical severity as an ordered outcome. All statistical analyses were performed using R version 4.4.3 (R Foundation for Statistical Computing, Vienna, Austria).

### 2.6 Ethics

The study was approved by the Institutional Research Ethics Committee of the Health Sciences Center at the University of Guadalajara (registry number CI-05925) and registered at ClinicalTrials.gov (NCT07408947). Given the retrospective nature of the study and the use of anonymized data, the requirement for informed consent was waived.

## 3 Results

**FIGURE 1:**
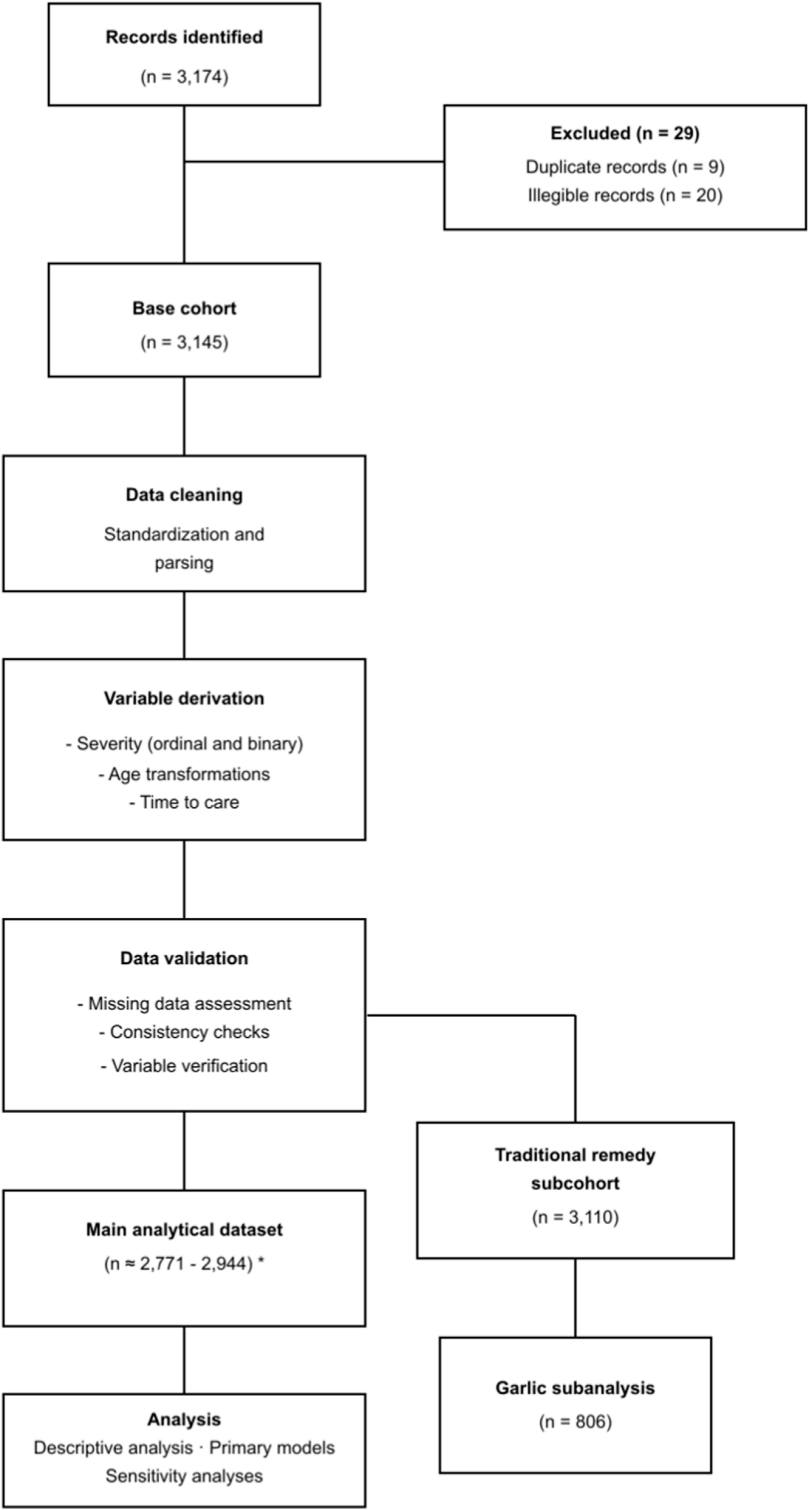
Flowchart of patient selection and analytical dataset construction. Following exclusion of duplicate and illegible records, eligible patients underwent data cleaning, variable derivation, and validation procedures. *The final sample size varied according to data completeness and the requirements of each analysis. The traditional remedy and garlic subcohorts were used for specific secondary analyses.

A total of 3,145 patient registries were included in the analysis (Figure 1). The median age was 23 years (IQR 12–38), and 57% of patients were female. The median body weight was 60 kg (IQR 44–75). Most scorpion stings occurred in the patient’s home (91%), and 43.2% involved the upper limbs. The median time from sting to medical care was 30 min (IQR 20–60). Patients in clinically vulnerable age groups (<10 years and >60 years) represented approximately 25% of the cohort (18.6% and 6.0%, respectively), with differences in severity distribution across age strata, including a higher proportion of severe cases among older adults.

**FIGURE 2:**
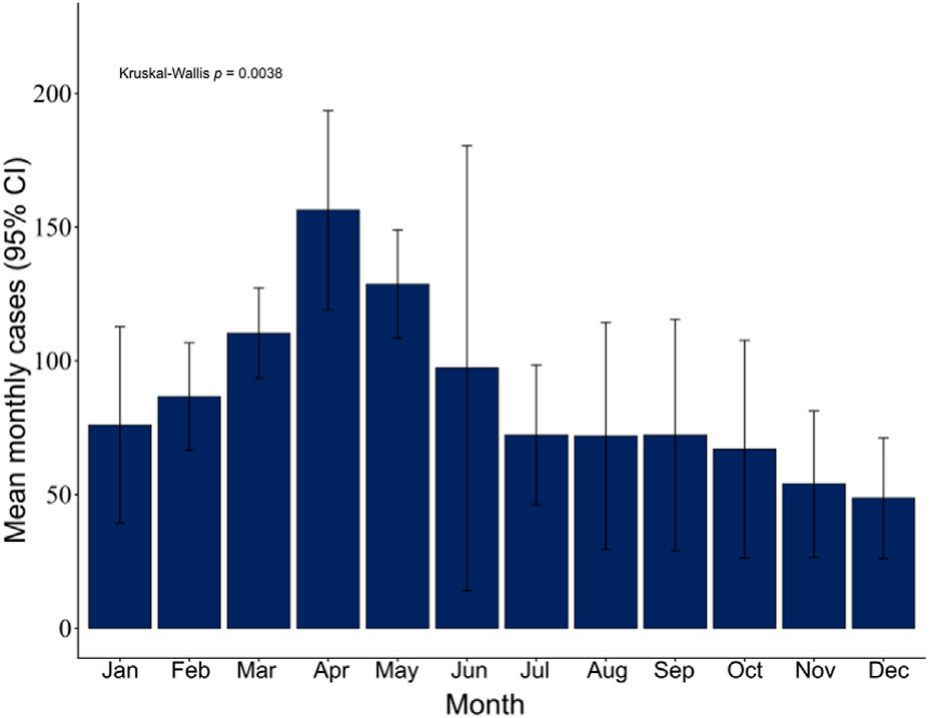
Mean monthly number of scorpion sting cases. Data are shown as monthly means with 95% confidence intervals, calculated from annual counts recorded between 2021 and 2025. Error bars indicate 95% confidence intervals. Monthly variation was assessed using the Kruskal–Wallis rank sum test, which showed significant differences across months (*p =* 0.004).

Monthly incidence showed significant variation throughout the year (Kruskal–Wallis rank sum test, *p =* 0.004). A clear seasonal pattern was observed, characterized by a progressive increase in cases from January to April, followed by a decline toward the end of the year. The highest monthly mean number of cases was observed during April (156.3 cases/month), followed by May (128.7 cases/month) and March (110.3 cases/month). In contrast, the lowest incidence occurred during December (48.7 cases/month) and November (54.0 cases/month) (Figure 2).

**FIGURE 3:**
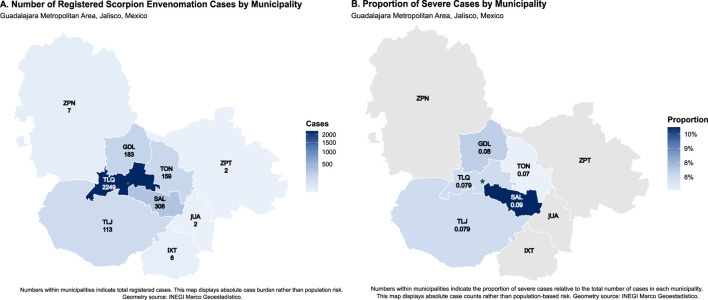
Geographic distribution of scorpion envenomation cases within the Guadalajara Metropolitan Area, Jalisco, Mexico (2021–2025). **(A)** Total number of registered cases by municipality. **(B)** Proportion of severe cases by municipality. Numbers shown in panel B indicate the proportion of severe cases relative to the total number of cases recorded in each municipality. Proportion estimates were calculated only for municipalities with ≥10 reported cases to avoid unstable estimates. The green star indicates the location of the study hospital. Maps represent hospital-treated cases and should not be interpreted as population incidence or population-based risk. Geometry source: INEGI Marco Geoestadístico. Abbreviations: GDL, Guadalajara; ZPN, Zapopan; TLQ, San Pedro Tlaquepaque; TLJ, Tlajomulco de Zúñiga; TON, Tonalá; SAL, El Salto; ZPT, Zapotlanejo; IXT, Ixtlahuacán de los Membrillos; JUA, Juanacatlán.

Most patients originated from San Pedro Tlaquepaque (n = 2,249), followed by El Salto (n = 308), Zapopan (n = 183), Guadalajara (n = 159), and Tlajomulco de Zúñiga (n = 113) (Figure 3). Fewer cases were identified in Tonalá (n = 6), Juanacatlán (n = 2), and Ixtlahuacán de los Membrillos (n = 2). Although most cases originated from San Pedro Tlaquepaque, the proportions of moderate-to-severe and severe envenomation were only slightly higher in some neighboring municipalities. Overall, clinical severity showed limited geographic variation across the Guadalajara Metropolitan Area. Proportion estimates were restricted to municipalities with at least 10 reported cases to avoid instability associated with very small sample sizes.

Prehospital use of at least one traditional remedy before presentation was reported by 849 patients (27%). Among these, oral milk consumption was the most frequently reported practice (77%, n = 657), followed by oral garlic use (30%, n = 257), topical application of sodium hypochlorite (5.5%, n = 47), and other remedies (11%, n = 91) including topical and oral herbs, other alimentary products, infusions, and non-standard preparations (Table 1). No deaths were recorded in this cohort.

**TABLE 1:** Baseline characteristics of the study population (N = 3,145).

Characteristic	N = 3,145^1^
Age (years)	23 (12, 38)
Sex	
Female	1,805 (57%)
Male	1,337 (43%)
Missing data	3 (0.1%)
Weight (kg)	60 (44, 75)
Location of sting	
Crop field	34 (1.1%)
School	23 (0.7%)
Factory	28 (0.9%)
Office	22 (0.7%)
Other	153 (4.9%)
Home	2,867 (91%)
Anatomical region of sting	
Head	163 (5.2%)
Two distinct sting sites	115 (3.7%)
Lower limb	836 (26.6%)
Upper limb	1,358 (43.2%)
Trunk	353 (11.2%)
Missing data	320 (10.2%)
Traditional treatment	
No	2,261 (72%)
Yes	849 (27%)
Missing data	35 (1.1%)
Time to care (min)	30 (20, 60)
Total antivenom vials	1.00 (0.00, 1.00)
Clinical classification	
Mild	1,010 (32%)
Moderate	1,876 (60%)
Severe	259 (8.2%)

1 Median (Q1, Q3); n (%).

The most frequently reported symptom at hospital presentation was local pain, recorded in 95.5% of patients (n = 3,002), followed by local paresthesia in 66.4% (n = 2,087) and a sensation of a foreign body in the throat in 40% of cases (n = 1,261). The frequency of other local, neurological, and systemic signs and symptoms is detailed in Table 2.

**TABLE 2:** Frequency of clinical signs and symptoms among patients with scorpion envenomation.

Symptom	Frequency (n)	Percentage (%)
Local pain	3,002	95.5
Paresthesias	2,087	66.4
Foreign body sensation in throat	1,261	40.1
Fasciculations	870	27.7
Sialorrhea	716	22.8
Oropharyngeal pruritus	663	21.1
Dysphagia	510	16.2
Restlessness	490	15.6
Dry mouth	478	15.2
Pruritus	466	14.8
Anxiety	429	13.6
Rhinorrhea	385	12.2
Conjunctival hyperemia	316	10.0
Epiphora	292	9.3
Headache	245	7.8
Crying	194	6.2
Tachycardia	184	5.9
Sneezing	138	4.4
Hypertension	138	4.4
Dyspnea	96	3.1
Nystagmus	91	2.9
Abdominal pain	56	1.8
Abdominal distension	35	1.1
Photophobia	19	0.6
Mydriasis	15	0.5
Dysarthria	14	0.4
Fever	13	0.4
Miosis	13	0.4
Vulvar pruritus	6	0.2

**FIGURE 4:**
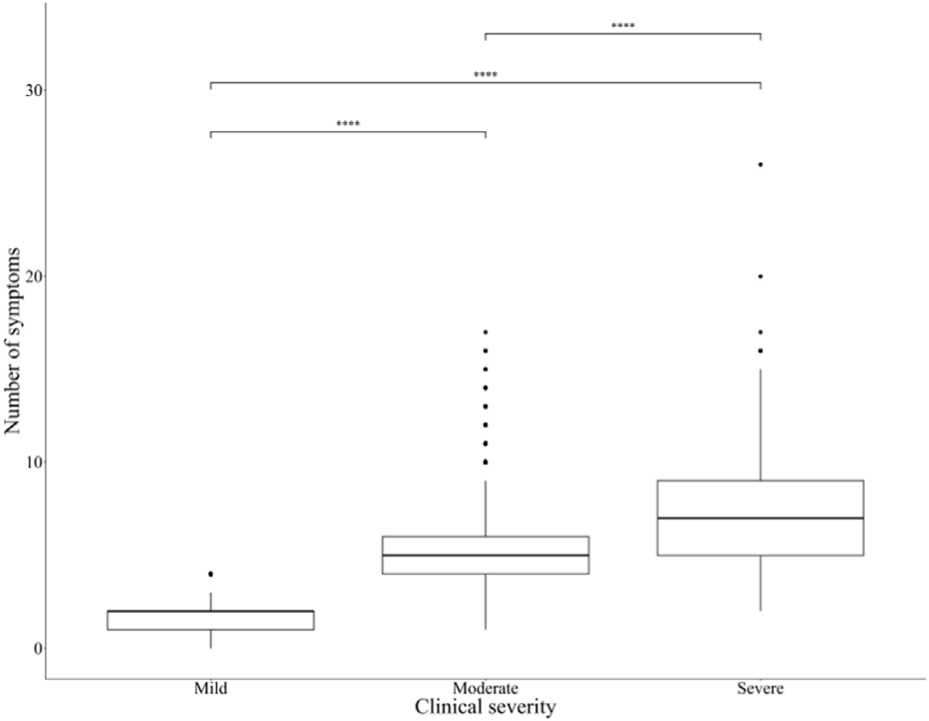
Symptom burden according to clinical severity of scorpion envenomation. Box plots represent the median (central line), interquartile range (box), and range (whiskers). Overall differences were evaluated using the Kruskal–Wallis test. Significance levels correspond to Holm-adjusted p values: *p ≤* 0.05 (*), *p ≤* 0.01 (**), *p ≤* 0.001 (***), and *p ≤* 0.0001 (****).

The median number of signs and symptoms per patient was 4 (IQR 2–6) in the overall population. When stratified by clinical severity, a progressive increase in the number of manifestations was observed across severity categories, with patients classified as moderate and severe presenting a higher symptom burden compared to those with mild envenomation (p < 0.001) (Figure 4).

**FIGURE 5:**
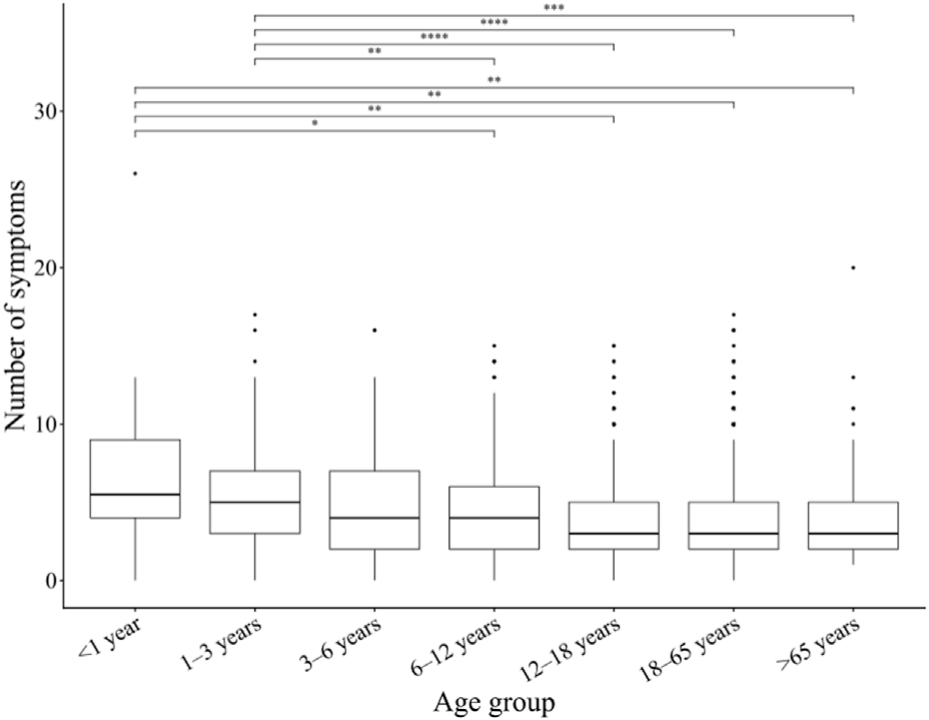
Distribution of the number of signs and symptoms by age group among patients with scorpion envenomation. Box plots represent the median (central line), interquartile range (box), and 1.5×IQR (whiskers); dots indicate outliers. Differences between groups were assessed using the Kruskal–Wallis test followed by Dunn’s *post hoc* test with Holm adjustment for multiple comparisons. Only statistically significant pairwise comparisons are show. Significance levels correspond to Holm-adjusted p values: *p ≤* 0.05 (*), *p ≤* 0.01 (**), *p ≤* 0.001 (***), and *p ≤* 0.0001 (****).

A weak inverse correlation was observed between age and the number of reported signs and symptoms (Spearman’s ρ = −0.085, p < 0.001). In *post hoc* analyses stratified by age groups, patients younger than 1 year presented a median of 5.5 symptoms (IQR 4–9), compared with 4 (IQR 2–6) among children aged 3–6 years and 3 (IQR 2–5) among patients aged 65 years or older (Figure 5).

Of the 3,145 patients included, 1,010 (32.1%) were classified as mild, 1,876 (59.6%) as moderate, and 259 (8.2%) as severe envenomation. Increasing clinical severity was associated with longer time to medical care and greater antivenom use (p < 0.001). Severity distribution varies according to age. Although median age was similar across severity categories (25 years (IQR 15–38) for mild cases, and 22 years (IQR 11–37) for moderate or severe cases) the distributions differed across groups (p < 0.001). Median body weight and time to care followed similar patterns across severity strata. Physician-registered sex was not associated with severity (Table 3).

**TABLE 3:** Baseline characteristics according to binary clinical severity classification.

Variable	Clinical severity		
	Mild N = 1,010	Moderate or severe N = 2,135	p-value^1^
Age (years)	25 (15, 41.8)	22 (11, 37)	<0.001
Sex	​	​	0.7
Female	575 (57%)	1,230 (58%)	​
Male	434 (43%)	903 (42%)	​
Weight (kg)	65 (50, 78)	60 (39, 74)	<0.001
Time to care (min)	30 (20, 50)	30 (20, 60)	<0.001
Total antivenom vials	0.00 (0.00, 0.00)	1.00 (1.00, 1.00)	<0.001
Traditional treatment	276 (27.3%)	573 (26.8%)	0.796
Oral garlic use	61 (6.0%)	196 (9.2%)	0.003

1 Continuous variables are presented as median (Q1–Q3) and categorical variables as n (%). Binary severity classification defined as mild (local manifestations only) versus moderate/severe (presence of systemic signs and symptoms). Group differences were assessed using the Kruskal–Wallis rank sum test for continuous variables and Fisher’s exact test for categorical variables.

In multivariable logistic regression analyses, longer time from sting to medical care was associated with higher odds of moderate or severe envenomation (adjusted odds ratio [aOR] 1.08 per hour increase, 95% CI 1.013–1.164; *p =* 0.027). In contrast, increasing age (aOR 0.772 per year, 95% CI 0.698–0.851; p < 0.001) and higher body weight (aOR 0.973 per 10 kg, 95% CI 0.961–0.984; p < 0.001) were associated with lower odds of increased clinical severity.

Overall use of traditional remedies was not associated with clinical severity in multivariable models (aOR 1.019, 95% CI 0.857–1.213; *p =* 0.833). In exploratory analyses of individual remedies, oral garlic use showed a positive association with moderate or severe envenomation (aOR 1.625, 95% CI 1.145 – 2.33; *p =* 0.007), whereas other remedies were not associated with severity (Table 4).

**TABLE 4:** Multivariable logistic regression analysis of factors associated with moderate or severe scorpion envenomation.

Variable	Adjusted OR	95% CI	p-value
Traditional treatment	1.019	0.857–1.213	0.833
Oral garlic use	1.625	1.145–2.33	0.007
Age (per year)	0.772	0.698–0.851	<0.001
Time to care (per hour)	1.08	1.013–1.164	0.027
Body weight (per 10 kg)	0.973	0.961–0.984	<0.001
Male sex	0.929	0.791–1.092	<0.370

Adjusted odds ratios (ORs) with 95% confidence intervals (CI) were estimated using multivariable logistic regression. Continuous variables are expressed per unit increase as indicated (age per year, time to care per hour increase, and body weight per 10 kg). The model was adjusted for age, sex, time to medical care, body weight, and oral garlic use.

Exploratory analyses were conducted to examine whether prehospital oral garlic use was associated with specific clinical manifestations at presentation. Patients reporting garlic consumption showed a higher frequency of upper pharyngeal symptoms compared with non-users, particularly a foreign body sensation in the pharynx (χ^2^ = 16.9; *p* < 0.001). Sneezing was also more frequently reported in this group, although the association was weaker and of borderline statistical significance (χ^2^ = 4.0; *p =* 0.046). No clear associations were observed between garlic use and other local, neurological, respiratory, or systemic manifestations, including sialorrhea, dysphagia, fasciculations, respiratory distress, cardiovascular signs, or severe neurological symptoms. These findings are presented as hypothesis-generating and should be interpreted cautiously given the potential for residual confounding and differences in symptom perception or reporting.

## 4 Discussion

In this cross-sectional study, we evaluated factors associated with clinical severity in scorpion envenomation in western Mexico. Longer time to medical care was consistently associated with greater clinical severity across models. In addition, patient characteristics such as younger age and lower body weight were also associated with more severe clinical presentation, reflecting differences in clinical presentation across individuals. Although these findings do not imply causality, they highlight patterns that may be relevant for clinical assessment and health system response.

The seasonal pattern observed in our study also agrees with reports from Colima, one of the regions with the highest incidence of scorpion stings in Mexico. Chowell et al. described a seasonal increase in sting incidence associated with warmer months and the rainy season, reporting higher activity during periods of elevated temperature and precipitation (Chowell et al., 2006). Similarly, subsequent analyses demonstrated that minimum temperature was one of the strongest climatological predictors of sting incidence, explaining a substantial proportion of temporal variability (Chowell et al., 2005). At the national level, Hernández-Muñoz et al. also documented a stable seasonal pattern, with incidence increasing from February to June, showing a slight decline during mid-summer, and decreasing during the winter months (Hernández-Muñoz et al., 2026). Comparable findings have also been reported in Argentina, where case frequency peaked during the warmest months of the year (December - January) and declined markedly during winter, as well as in Tunisia, where admissions to pediatric intensive care units showed a pronounced seasonal distribution, with 85.4% of admissions occurring during summer (Suasnábar et al., 2022; Elleuch et al., 2026). Together, these observations further support the influence of environmental temperature on scorpion activity and human exposure.

These observations are consistent with ecological patterns reported across endemic regions worldwide. In Turkey, both average and minimum temperature showed strong positive correlations with scorpion sting incidence, whereas relative humidity was negatively associated with case frequency (İşkorur et al., 2026). In Iran, higher temperatures, evaporation, and sunshine duration were associated with increased sting incidence, while rainfall and humidity were associated with lower incidence (Ghorbani et al., 2021). Likewise, in Brazil, mean annual temperature was identified as a major environmental predictor of scorpion sting incidence, whereas precipitation showed no significant association (Vilarinho et al., 2023).

Several mechanisms may explain this seasonal behavior. Scorpions are nocturnal arthropods that generally occupy relatively small areas and depend on protected microhabitats such as rocks, tree bark, fallen logs, and soil refuges. Their activity patterns are strongly influenced by environmental conditions, particularly temperature, which has been associated with increased surface activity and greater detectability in several species. Rainfall may also contribute indirectly by flooding natural refuges and burrows, forcing scorpions to seek alternative shelter in and around human dwellings. This hypothesis has been proposed previously in endemic regions of Mexico and is supported by the temporal coincidence between rainfall peaks and increased sting incidence (Chowell et al., 2005; Chowell et al., 2006; Morales-Martínez et al., 2025).

From a public health perspective, the persistence of this seasonal pattern highlights the potential value of anticipatory prevention strategies. Recent ecological niche models further suggest that climate change may alter the distribution of medically important scorpion species, potentially increasing the public health burden in some regions through shifts in habitat suitability driven by temperature and precipitation patterns (Freitas Barroso et al., 2025). Community education campaigns, reinforcement of environmental control measures, and optimization of antivenom distribution could be implemented before the months of highest incidence, thereby improving preparedness during predictable periods of increased risk.

Only limited geographic variation in the proportion of moderate-to-severe envenomation was observed across municipalities within the Guadalajara Metropolitan Area. Although slight differences were present, the overall distribution of clinical severity was broadly similar across municipalities. These findings suggest that, within this metropolitan setting, factors related to healthcare access, travel distance, and other contextual determinants may have a relatively modest influence on clinical severity. Nevertheless, geographic patterns of scorpion envenomation may still be shaped by multiple interacting factors, including exposure patterns, local scorpion fauna, social vulnerability, and healthcare accessibility (Siqueira et al., 2024).

The observed gradient in symptom burden across severity categories supports the internal consistency of the clinical classification, as greater severity was accompanied by a broader spectrum of systemic involvement. These findings are consistent with prior clinical descriptions of scorpion envenomation, in which the neurotoxic syndrome is defined by the concurrent presence of multiple manifestations, including neuromotor, oculomotor, and respiratory abnormalities, rather than by isolated features (Boyer et al., 2009). In this context, the number of signs and symptoms may serve as a proxy for the peak clinical expression of envenomation, reflecting the intensity of systemic toxin activity at its maximum. Thus, progression from mild to moderate and severe envenomation appears to represent not only the emergence of specific systemic manifestations but also an accumulation of concurrent clinical features, supporting a continuum model of disease severity rather than discrete categories.

Age was inversely associated with clinical severity in our analysis, a pattern that has also been described in previous studies. In particular, Jean-Philippe et al. reported that nearly 90% of fatalities from scorpion envenomation occurred in patients younger than 10 years (Chippaux et al., 2020). Similar age-related patterns have recently been reported in independent pediatric cohorts from Israel and Argentina, where children with more severe envenomation were significantly younger than those with mild presentations. Comparable findings have also been reported in Morocco, where patients younger than 1 year of age presented the most severe cases and the highest mortality rates, as well as in Tunisia, where children younger than 5 years accounted for 66% of admissions to pediatric intensive care units (Elleuch et al., 2026; Darkaoui et al., 2024; Kestenbom et al., 2026). These results support the role of age as an important determinant of clinical expression following scorpion envenomation, and are also in line with prior reports describing differences in clinical outcomes according to host characteristics such as body size, weight, and baseline health status (Chippaux et al., 2020). These observations suggest consistent age-related patterns in the clinical presentation of envenomation and may reflect differences in physiological response, body size, and effective venom concentration across age groups.

Our findings regarding the association between clinical severity and delay in medical care are consistent with those reported by Chowell et al., who described a similar pattern between patient delay and envenomation severity, with longer intervals before accessing health services among severely affected individuals (Chowell et al., 2006). Supporting this interpretation, a recent pediatric study from Brazil identified delays of ≥3 h between the sting and medical care as an independent predictor of hospitalization, suggesting that delayed access to care may contribute to worse clinical outcomes (Tonin dos Santos et al., 2025). These observations may reflect differences in healthcare access, symptom recognition, or care-seeking behavior. Alternatively, patients with more severe symptoms may experience barriers or delays before reaching care, which cannot be disentangled in a cross-sectional design.

Previous reports have indicated that up to 90% of patients stung by scorpions resort to home remedies prior to seeking medical attention or in place of it (Lagunas-Flores and Lagunas-Jaimes, 2009). The use of traditional remedies before seeking medical attention is not unique to Mexico and has been documented across several endemic regions. Studies from Pakistan have shown frequent use of plant-based remedies and traditional healers, highlighting the cultural and contextual dimensions of prehospital care-seeking behavior (Ahsan et al., 2024). In the present study, the use of traditional remedies as a composite exposure was not associated with increased clinical severity in multivariable models. In exploratory analyses examining individual practices, oral garlic use showed a positive association with more severe envenomation.

The mechanism underlying these observations remains uncertain. Raw garlic contains sulfur compounds that may irritate mucus membranes. Chemical burns, severe allergic or contact irritant dermatitis of topical garlic used as a home remedy have been described (Chiriac et al., 2017). These effects could plausibly produce or amplify upper aerodigestive symptoms—such as a foreign body sensation in the pharynx or sneezing—that overlap with early manifestations of envenomation, potentially influencing symptom perception or reporting. In this context, the observed association may reflect a non-specific irritative effect rather than a direct contribution to systemic toxicity. Alternatively, garlic use may be a marker of differences in care-seeking behavior, including delayed presentation or attempts at symptom management prior to medical evaluation, which could modify the clinical picture at the time of assessment.

Together, these findings underscore the importance of distinguishing between traditional practices as a general phenomenon and specific behaviors when interpreting patterns of clinical presentation.

The clinical signs and symptoms observed in this cohort were largely consistent with those reported by Chowell et al., who identified local pain, local paresthesia, pruritus, sensation of a foreign body in the throat, sialorrhea, and restlessness as the most frequent manifestations of scorpion envenomation (Chowell et al., 2006). Differences in symptom frequencies were observed between studies. In our cohort, pruritus was reported in 14% of patients (n = 466), compared with 54.3% in the series described by Chowell et al. In addition, tongue fasciculations, present in 27.7% of our patients (n = 870), but were not reported in that series, this finding has been described in other reports of scorpion envenomation involving cranial nerve manifestations (Quan et al., 2019). Nystagmus was observed in only 2.9% of patients in our cohort (n = 91), markedly lower than the prevalences reported in other series, including those by Quan et al. (>92.3%), Gateau et al. (57.6%), and Klotz et al. (63.2%). (Klotz et al., 2021; Quan et al., 2019). As reported in Brazil, differences in symptom frequencies between studies may also reflect species-specific venom effects and regional differences in scorpion fauna (Takehara et al., 2023).

The median number of signs and symptoms observed in our cohort was similar to that reported by Gateau et al. and showed a weak inverse correlation with age (Spearman’s ρ = −0.085, p < 0.001), indicating a slight decrease in symptom burden with increasing age (Gateau et al., 1994). This pattern is consistent with descriptive analyses across age groups, in which younger patients presented a higher number of clinical manifestations. These observations are not fully aligned with national guidelines that consider older patients to be at comparable risk to children (CENETEC. Guía de Práctica Clínica, 2015). Notwithstanding that older patients present fewer clinical manifestations, lethality among patients ≥50 years (0.014%) is 6.4-fold higher than in the ≥10–49 years age group (0.002%). Across all age groups, lethality is highest in those aged <1 year (0.4%), 1–4 years (0.1%) and 5–9 years (0.02%). Rather than contradicting these recommendations, our results suggest that risk may vary across age groups and should be interpreted within a broader clinical context (Hernández-Muñoz et al., 2026).

The observed variation in severity according to age may reflect differences in body size and weight across patient groups. Prior studies have reported higher serum levels of *Centruroides* venom in younger patients, suggesting potential differences in venom distribution according to age (Osnaya- Romero et al., 2016).

In Mexico, national clinical guidelines recommend antivenom administration based on predefined risk factors, including the use of higher antivenom doses in children and older adults, given their increased probability of complications (CENETEC. Guía de Práctica Clínica, 2015; Silanes, 2023). In our cohort, clinical severity varied across age groups, with both younger and older patients represented among severe cases. Our findings suggest that severity and mortality may not follow identical age-related patterns. While younger patients appear more likely to develop severe clinical manifestations, older adults may remain vulnerable to adverse outcomes because of reduced physiological reserve and underlying comorbidities. Similar concerns regarding age-dependent vulnerability have been reported in other endemic regions (Darkaoui et al., 2024).

These findings highlight the relevance of considering clinical features at presentation—such as the type and number of signs and symptoms, as well as time to medical care—when interpreting severity, in addition to age-based risk categories. Currently, both in the United States and Mexico, alternative management approaches are being explored to reduce antivenom dosing while maintaining clinical effectiveness, with the aim of improving accessibility and cost-effectiveness of treatment (Quan et al., 2019; Villa-Manzano et al., 2020; Sinha et al., 2016).

Taken together, composite use of traditional remedies was not associated with clinical severity in adjusted exploratory models. In contrast, oral garlic use showed an exploratory association with greater severity, which may reflect differences in care-seeking behavior, symptom perception, exposure, misclassification, or residual confounding. Age and body weight were also associated with severity across models, reflecting variation in clinical presentation across patient groups.

The generalizability of our findings should be interpreted in the context of the ecological and healthcare setting in which the study was conducted. This study took place in an emergency department in western Mexico, where scorpion envenomation is endemic and primarily caused by species of the genus *Centruroides*. Therefore, our results are likely generalizable to regions with a similar epidemiological profile, particularly areas in Mexico and the Southern United States where *Centruroides* species predominate.

In these settings, the observed associations between time to care, patient characteristics, and clinical severity may reflect shared pathophysiological mechanisms and comparable patterns of envenomation. However, caution is warranted when extrapolating these findings to regions with different scorpion fauna, healthcare systems, or access to antivenom, as variations in venom composition, clinical management, and care-seeking behavior may influence disease presentation and outcomes.

Additionally, as this was a single-center, hospital-based study using routinely collected clinical data, the findings may not fully represent cases managed outside referral centers, or those not seeking medical attention. Nonetheless, the large sample size and use of standardized national surveillance definitions support the applicability of our results within comparable endemic regions.

This study includes a large sample size and reflects real-world clinical practice in a high-burden setting. The use of standardized epidemiological records allowed consistent data collection, and multiple sensitivity analyses supported the robustness of the findings. Although scorpion envenomation represents a major public health burden, to our knowledge, few studies have systematically evaluated factors associated with clinical severity in patients with scorpion envenomation, while accounting for the use of home remedies and adjusting for potential confounders through multivariable analysis.

The study has some limitations. First, its retrospective design limits causal inference, and residual confounding cannot be fully excluded. Second, data on venom load and the precise timing of symptom onset were not available, which may have limited a more detailed characterization of exposure–response relationships. In addition, information on prehospital practices relied on clinical records and patient or caregiver reports, which may be subject to misclassification or recall bias. These sources of bias may lead to overestimation or underestimation of associations, particularly for self-reported exposures such as traditional remedy use. Missing data were handled using complete-case analysis, with similar findings observed in sensitivity analyses using multiple imputation, although some degree of imprecision may remain. The study population was derived from a referral center and may not fully represent patients without access to hospital-based care, which may limit generalizability to other settings. Future prospective studies are needed to better characterize temporal relationships and to evaluate biological mechanisms, including direct measurement of venom exposure.

Overall, these findings contribute to the understanding of clinical presentation in scorpion envenomation and may inform clinical assessment, triage, and public health strategies aimed at reducing morbidity.

## 5 Conclusion

In this large cohort of patients with scorpion envenomation from western Mexico, we identified consistent associations between clinical severity and patient characteristics, including younger age, lower body weight, and longer time to medical care. A clear seasonal pattern of incidence was observed, with cases increasing during warmer months, supporting the influence of environmental factors on scorpionism dynamics. Although the overall use of traditional remedies was not associated with severity, exploratory analyses suggested that oral garlic consumption may influence symptom perception and clinical presentation. Together, these findings provide a comprehensive characterization of contemporary scorpion envenomation in an endemic region and may inform risk stratification, clinical assessment, public health preparedness, and educational strategies aimed at reducing morbidity.

## Data Availability

The de-identified raw data supporting the conclusions of this article will be made available by the authors upon reasonable request to the corresponding author (johana.banos@academicos.udg.mx). Data will be shared with researchers who provide a methodologically sound proposal, subject to approval by the study investigators and the signing of a data access agreement.
